# The *yad* and *yeh* fimbrial loci influence gene expression and virulence in enterohemorrhagic *Escherichia coli* O157:H7

**DOI:** 10.1128/msphere.00124-24

**Published:** 2024-06-21

**Authors:** Laura A. Gonyar, Amber B. Sauder, Lindsay Mortensen, Graham G. Willsey, Melissa M. Kendall

**Affiliations:** 1 Department of Microbiology, Immunology, and Cancer Biology, University of Virginia School of Medicine, Charlottesville, Virginia, USA; 2 Department of Microbiology and Molecular Genetics, Larner College of Medicine, University of Vermont, Burlington, Vermont, USA; The University of Iowa, Iowa, Iowa, USA

**Keywords:** EHEC, fimbriae, virulence regulation, virulence determinants, LEE

## Abstract

Fimbriae are essential virulence factors for many bacterial pathogens. Fimbriae are extracellular structures that attach bacteria to surfaces. Thus, fimbriae mediate a critical step required for any pathogen to establish infection by anchoring a bacterium to host tissue. The human pathogen enterohemorrhagic *Escherichia coli* (EHEC) O157:H7encodes 16 fimbriae that may be important for EHEC to initiate infection and allow for productive expression of virulence traits important in later stages of infection, including a type III secretion system (T3SS) and Shiga toxin; however, the roles of most EHEC fimbriae are largely uncharacterized. Here, we provide evidence that two EHEC fimbriae, Yad and Yeh, modulate expression of diverse genes including genes encoding T3SS and Shiga toxin and that these fimbriae are required for robust colonization of the gastrointestinal tract. These findings reveal a significant and previously unappreciated role for fimbriae in bacterial pathogenesis as important determinants of virulence gene expression.

**IMPORTANCE:**

Fimbriae are extracellular proteinaceous structures whose defining role is to anchor bacteria to surfaces. This is a fundamental step for bacterial pathogens to establish infection in a host. Here, we show that the contributions of fimbriae to pathogenesis are more complex. Specifically, we demonstrate that fimbriae influence expression of virulence traits essential for disease progression in the intestinal pathogen enterohemorrhagic *Escherichia coli*. Gram-positive and Gram-negative bacteria express multiple fimbriae; therefore, these findings may have broad implications for understanding how pathogens use fimbriae, beyond adhesion, to initiate infection and coordinate gene expression, which ultimately results in disease.

## INTRODUCTION

Fimbriae are important virulence traits common to Gram-negative and Gram-positive bacterial pathogens (1). Fimbriae play an essential role during infection by anchoring a pathogen to host tissue and preventing displacement from host fluxes or peristalsis (1, 2). Moreover, fimbrial adhesion enables pathogens to effectively deploy their distinct arsenals of virulence traits, including type III secretion systems (T3SS), effectors, or toxins (i.e., references 3–9). The established role of fimbriae in this process is strictly to mediate attachment (1). However, the question of whether fimbriae play additional roles in pathogenesis or whether fimbriae contribute directly to expression of factors important for disease progression has not been fully investigated.

Enterohemorrhagic *Escherichia coli* (EHEC) is a foodborne pathogen that causes major outbreaks of hemorrhagic colitis and hemolytic uremic syndrome (HUS) throughout the world (10). The canonical virulence factors of EHEC are Shiga toxin and the locus of enterocyte effacement (LEE). Shiga toxin inhibits mammalian protein synthesis and causes HUS, seizures, cerebral edema, and/or coma (11). The LEE encodes a T3SS and effectors that result in the formation of attaching and effacing (AE) lesions on colonic epithelial cells (12–14). AE lesions are characterized by intimate attachment of EHEC to enterocytes, effacement of the microvilli, and rearrangement of the underlying cytoskeleton, which results in formation of a pedestal-like structure beneath the bacterium. EHEC also encodes 16 fimbrial loci (15, 16) that are thought to contribute to tissue tropism and pathogenesis. EHEC fimbriae may be important in the early stages of EHEC colonization of the gastrointestinal (GI) tract that precede intimate attachment (17). However, the roles of fimbriae in EHEC pathogenesis are largely uncharacterized. This is due in part to the lack of understanding of environmental cues that promote expression of these fimbriae *in vitro* (18–20). We previously reported that EHEC exploits ethanolamine (EA), an abundant metabolite in the GI tract, as a signal to activate expression of virulence traits, including expression of fimbriae (21, 22). Here, we show that two of these fimbriae, Yad and Yeh, modulate extensive and distinct transcriptional changes in EHEC, including expression of the LEE and Shiga toxin. Moreover, these fimbriae promote EHEC colonization of the mammalian GI tract.

## RESULTS

### Yad and Yeh are chaperone/usher (CU) fimbriae

**Fig 1:**
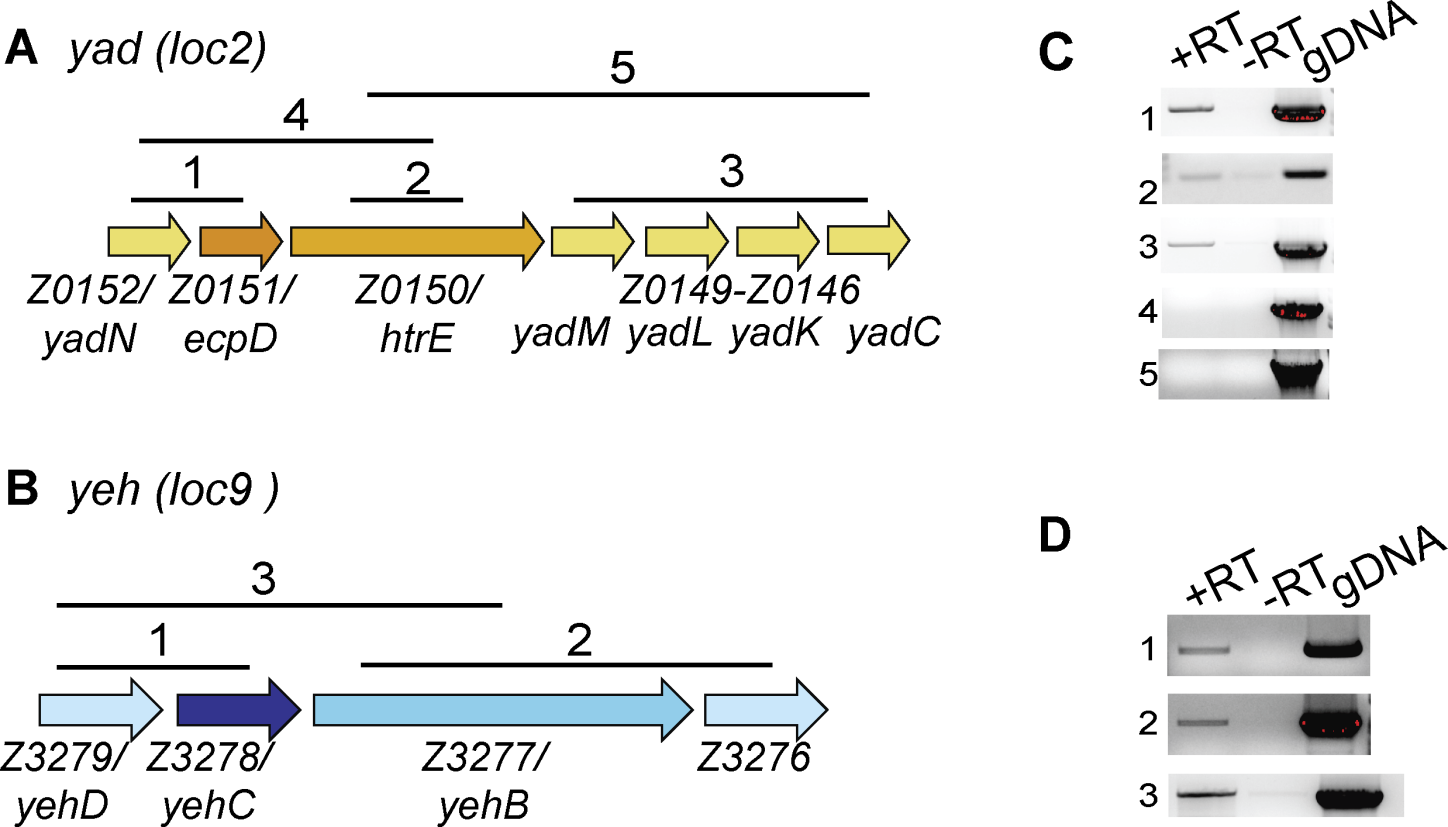
Organization and expression of genes encoded in the *yad* and *yeh* fimbrial loci. (**A**) Schematic representation of the *yad* genetic locus showing the predicted fimbrial subunits (yellow), usher (light orange), and chaperone (dark orange). Lines with numbers indicate regions amplified and correspond to PCR amplicons shown in panel **C**. (**B**) Schematic representation of the *yeh* genetic locus showing the predicted fimbrial subunits (light blue), usher (blue), and chaperone (purple). Lines with numbers indicate regions amplified and correspond to PCR amplicons shown in panel **D**. (**C**) RT-PCR of the *yad* locus. (**D**) RT-PCR of the *yeh* locus. For panels **C** and **D**, gDNA was used as a positive control, and a reaction without RT was used as a negative control.

In EHEC, EA promotes expression of 14 out of 16 fimbrial loci (21). Of these, preliminary observations suggested that the *yad* locus was most highly expressed compared to other loci when EHEC was grown in EA-supplemented media. Additionally, we measured the largest-fold change in *yeh* expression during EHEC growth in media with EA compared to growth in media without EA (21). These observations provided the rationale to study the contributions of Yad and Yeh to EHEC virulence in more detail. The Yad (*Z0146-Z0152;* locus 2) and Yeh (*Z3276-Z3279*; locus 9) fimbriae belong to the γ_4_ class of CU fimbriae (23). The minimal components of CU fimbriae include a major subunit, a chaperone that facilitates folding of fimbrial subunits in the periplasm, and an usher that serves as an assembly platform in the outer membrane of the bacterial cell; however, CU fimbriae may also encode additional subunits that contribute to binding specificity (23). In addition to the core fimbrial genes, the *yad* fimbrial locus contains four minor subunit genes, and the *yeh* fimbrial locus contains two subunit genes (Fig. 1A and B). These fimbrial loci are partially conserved between EHEC, other pathogenic *E. coli*, as well as non-pathogenic *E. coli* strains (19, 24). When constitutively expressed from non-native promoters, Yad and Yeh form functional fimbriae structures that contribute to *E. coli* K-12 adherence to abiotic surfaces (18); however, expression of these loci from the native promoter has not been demonstrated. Here, we confirmed that all the genes carried in *yad* and *yeh* fimbrial loci are expressed from the respective native promoters in EHEC. Moreover, reverse transcription PCR (RT-PCR) revealed that the *yad* locus is expressed as three transcriptional units (Fig. 1C), and the *yeh* locus is expressed as one transcript (Fig. 1D).

### CsrA promotes YehD expression

**Fig 2:**
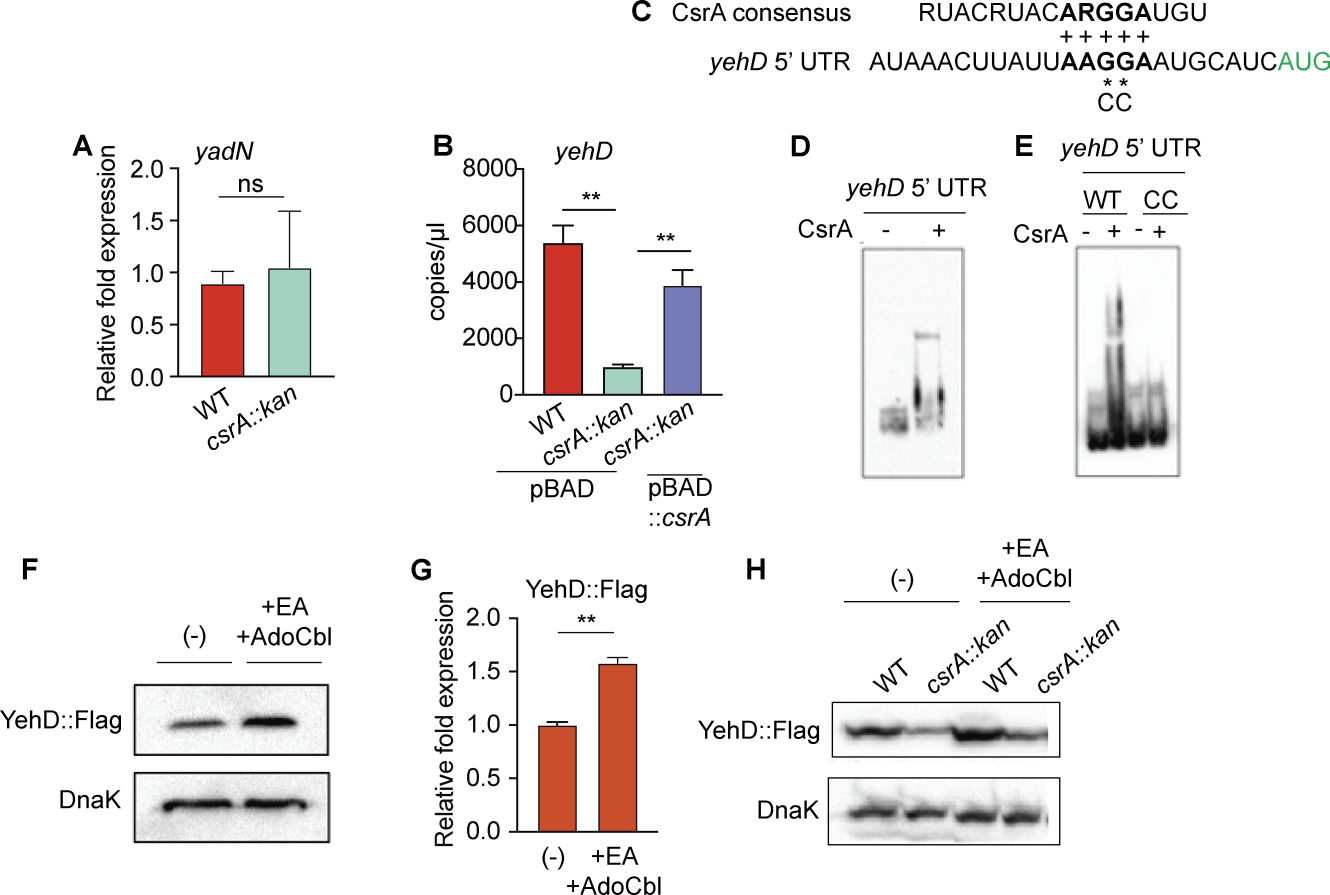
CsrA promotes *yehD* expression. (**A**) RT-qPCR of *yadN* in WT and *csrA::kan*. (**B**) RT-qPCR of *yehD* in WT with empty vector, *csrA::kan* with empty vector, and *csrA* complemented strains (rescued with plasmid complementation of *csrA*). (**C**) CsrA consensus binding site aligned with the *yeh* 5′ untranslated region (UTR). The critical residues for CsrA binding are bolded and the *yeh* start codon is green. (**D**) Electrophoretic mobility shift assay showing binding of CsrA to *yeh* 5′UTR and (**E**) dependence on the GG nucleotides. (**F**) Western blot of YehD::FLAG in WT grown in medium without or with 10 mM EA and 150 nM B_12_ supplementation. DnaK is used as a loading control. (**G**) Quantification of panel **F**. (**H**) Western blot of YehD-3×FLAG in WT or *csrA::kan* without or with 10 mM EA and 150 nM B_12_ supplementation. DnaK is used as a loading control. For panels **A**, **B**, **F**, **G**, and **H**, *n* = 3. Bars indicate the average and error bars represent the SEM. **P < 0.01.

The carbon storage regulatory protein CsrA binds RNA to regulate bacterial gene expression (25). CsrA has been reported to negatively regulate *yad* and *yeh* gene expressions in EHEC strain EDL933 (26). EHEC exhibits strain to strain variation in gene expression; therefore, we examined the effect of CsrA on *yad* and *yeh* expressions in EHEC strain 86-24. Because CsrA is an essential protein, we constructed a strain in which CsrA activity is reduced by inserting a kanamycin resistance cassette at codon 51 (27) (generating *csrA::kan*) and then assessed expression of *yadN* and *yehD,* the first gene in the respective locus. Expression of *yadN* was similar in wild-type (WT) and *csrA::kan* strains (Fig. 2A). Notably, *yehD* expression was significantly decreased in *csrA::kan* compared to WT, and this defect could be rescued by complementation (Fig. 2B). These data indicate that CsrA does not play a major role in *yadN* expression, but that CsrA is a positive regulator of *yehD* expression. These findings differ from CsrA regulation in EDL933 (26), which may be due to strain variation as well as growth conditions used in the assays.

Because the CsrA recognition motif RUACARGGAUGU (28) is nearly 100% conserved in the *yehD* 5′ untranslated region (5′UTR) (Fig. 2C), we performed protein-RNA electrophoretic mobility shift assays (EMSAs) to test whether CsrA directly regulates *yehD* expression. Upon addition of CsrA, we observed a shift in the *yeh* mRNA (Fig. 2D), which could be disrupted by mutation of two highly conserved CsrA binding residues GG to CC (Fig. 2E). These data identify CsrA as a direct regulator of fimbrial gene expression in EHEC.

To further characterize EA- and CsrA-dependent regulation of *yeh* expression, we generated a chromosomal *yehD::flag* gene and assessed YehD::Flag levels in EHEC grown without or with EA and adenosylcobalamin (AdoCbl), which is required for EA-dependent signaling and metabolism (29). In agreement with the transcriptional data (21), supplementation with EA and AdoCbl resulted in significantly increased Yeh::FLAG expression compared to growth without supplementation (Fig. 2F and G); however, these molecules were not required for CsrA-mediated YehD regulation (Fig. 2H). CsrA can affect transcript stability as well as translation to regulate target gene expression (30); future work will determine how CsrA controls *yehD* expression.

### Yad and Yeh differentially modulate expression of diverse genes in EHEC

**Fig 3:**
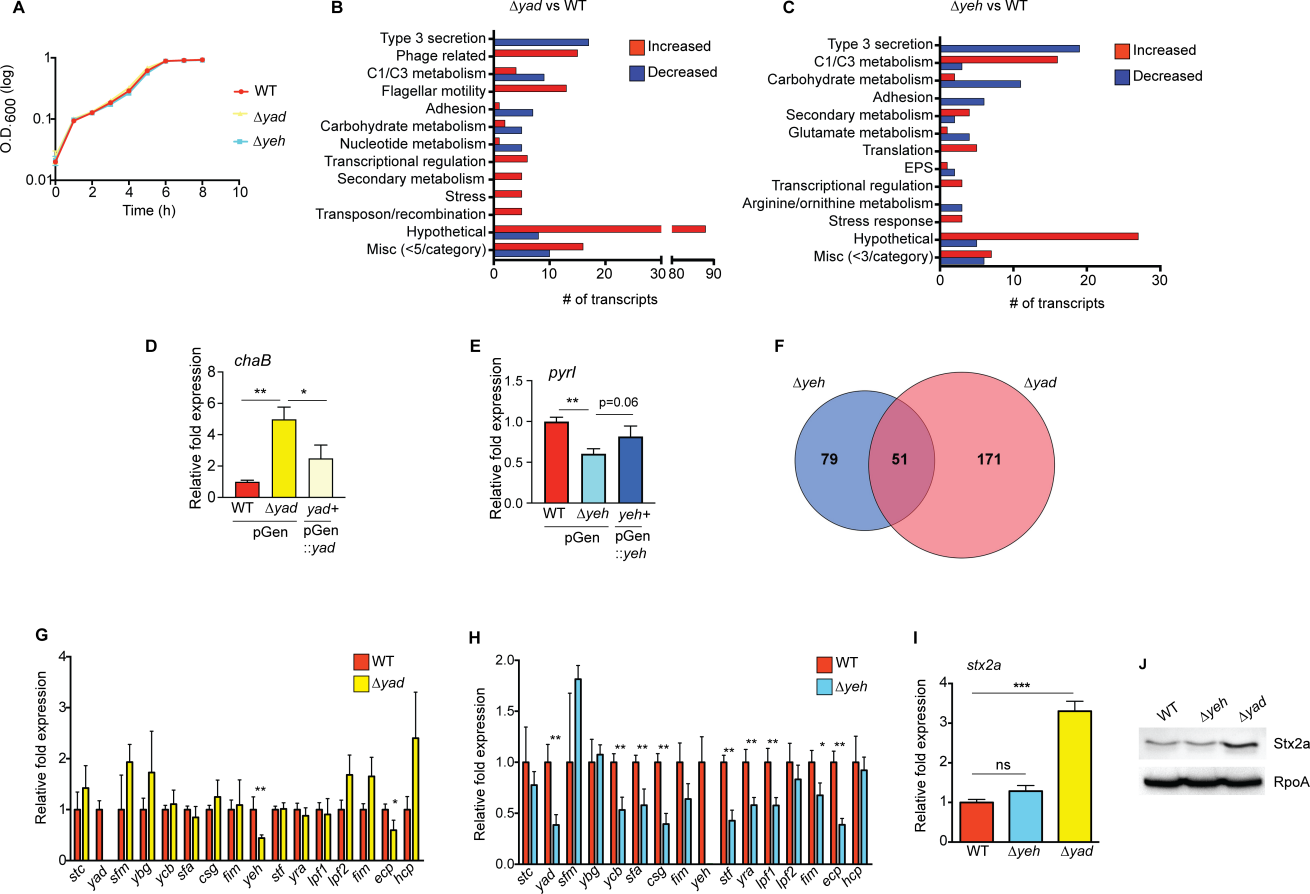
Yad and Yeh influence expression of diverse genes in EHEC. (**A**) *In vitro* growth curve of WT EHEC, the ∆*yad*, and the ∆*yeh* strains grown planktonically. Each data point shows the average of three biological replicates. Error bars represent the geometric mean ± standard deviation (SD). (**B**) Bar graph categorizing *E. coli* genes differentially expressed in the ∆*yad* strain compared to WT. (**C**) Bar graph categorizing *E. coli* genes differentially expressed in the ∆*yeh* strain compared to WT. (**D**) RT-qPCR of *chaB* in WT with empty vector, ∆*yad* with empty vector, *yad*+ (rescued with plasmid complementation of *yad*). (**E**) RT-qPCR of *pyrI* in WT with empty vector, ∆*yeh* with empty vector, *yeh*+ (rescued with plasmid complementation of *yeh*). (**F**) Venn diagram showing the number of overlapping genes between the ∆*yad* and ∆*yeh* strains. (**G**) RT-qPCR of one gene from each fimbrial locus in the WT and ∆*yad* strains. Loci are labeled according to the major subunit gene of each locus. (**H**) RT-qPCR of one gene from each fimbrial locus in the WT and ∆*yeh* strains. (**I**) RT-qPCR of *stx2a* in the WT, ∆*yeh*, and ∆*yad* strains. (**J**) Representative Western blots of Stx2a and RpoA (loading control) from cell lysates of WT, ∆*yeh*, and ∆*yad*. For panels **C**, **D**, **F**, **G**, and **H**, *n* = 3; error bars represent the geometric mean ± SD. *, *P* ≤ 0.05; **, *P* ≤ 0.005; ***, *P* ≤ 0.0005; ns, *P* > 0.05 (indicated in panel **H**).

Adherence can provide a physical stimulus that reprograms transcriptional networks, including changes in virulence expression in bacterial pathogens, through a process termed mechanosensing (31–37). Moreover, bacterial aggregation mediated by synthetic and native adhesins differently modified transcription in *E. coli* K-12 (38). To examine whether Yad and/or Yeh influence EHEC gene expression, we generated deletions of the entire *yad* or *yeh* loci, creating strains ∆*yad* and ∆*yeh*, and confirmed that these deletions did not affect EHEC growth (Fig. 3A). To investigate whether Yad and Yeh influence EHEC gene expression, we performed a microarray using the GeneChip *E. coli* Genome 2.0 array that includes approximately 10,000 probe sets for all genes present in the following four strains of *E. coli*: K-12 lab strain MG1655, uropathogenic strain CFT073, O157:H7 enterohemorrhagic strain EDL933, and O157:H7 enterohemorrhagic strain Sakai. For this assay, RNA was purified from planktonically grown WT EHEC strain 86-24, the ∆*yad* strain, or the ∆*yeh* strain. The transcriptomic studies revealed that in the ∆*yad* strain, 222 genes were differentially expressed compared to WT, whereas in the ∆*yeh* strain, 130 genes were differentially expressed compared to WT. Both fimbriae affected expression of genes involved in T3S, metabolism, transcriptional regulation, and stress responses (Fig. 3B through E, and data described below). Of the differentially expressed genes, only 51 genes were common between the fimbrial deletion strains (Fig. 3F).

Bacteria often encode a diverse repertoire of fimbriae, and expression of a fimbrial locus may enhance or repress expression of another surface adhesin (39–45). This coordinated expression, or cross-talk, between fimbrial loci may be important to enhance binding to host tissue and/or modulate host responses (44). The transcriptomic data indicated that Yad and Yeh primarily function to positively influence expression of adhesins (Fig. 3B and C). To further examine the impact of Yad and Yeh on expression of other adhesins, we performed RT-qPCR and measured expression of one gene from each fimbrial locus in the WT, ∆*yad*, and ∆*yeh* strains. Expression of *yeh* and *hcp* genes was decreased in ∆*yad* compared to WT (Fig. 3G), indicating Yad positively influenced expression of these loci. Yeh had a more extensive role in altering fimbrial expression, as expression of genes in nine fimbrial loci, including the *yad* loci, was decreased in the ∆*yeh* strain compared to WT (Fig. 3H). These findings suggest that positive cross-talk exists between the Yad and Yeh fimbriae and that at least a subset of EHEC fimbriae functions synergistically.

Additionally, bacteriophages have contributed to the evolution and pathogenesis of EHEC (15, 16). The array data revealed that phage-related genes were increased in the ∆*yad* strain compared to WT (Fig. 3B). The genes encoding Shiga toxin are located in intact or partial genomes of prophages from the lambda family that are integrated into the bacterial chromosome (46, 47). In agreement with the transcriptomic analyses, expression of *stx2a,* which encodes Shiga toxin, was significantly increased in the ∆*yad* strain compared to both the WT and ∆*yeh* strains at the transcriptional and translational levels (Fig. 3I and J), indicating that Yad influences Shiga toxin production. Collectively, these data reveal that Yad and Yeh play extensive, but distinct, roles in altering EHEC gene expression.

### Yad and Yeh promote LEE expression and AE lesion formation

**Fig 4:**
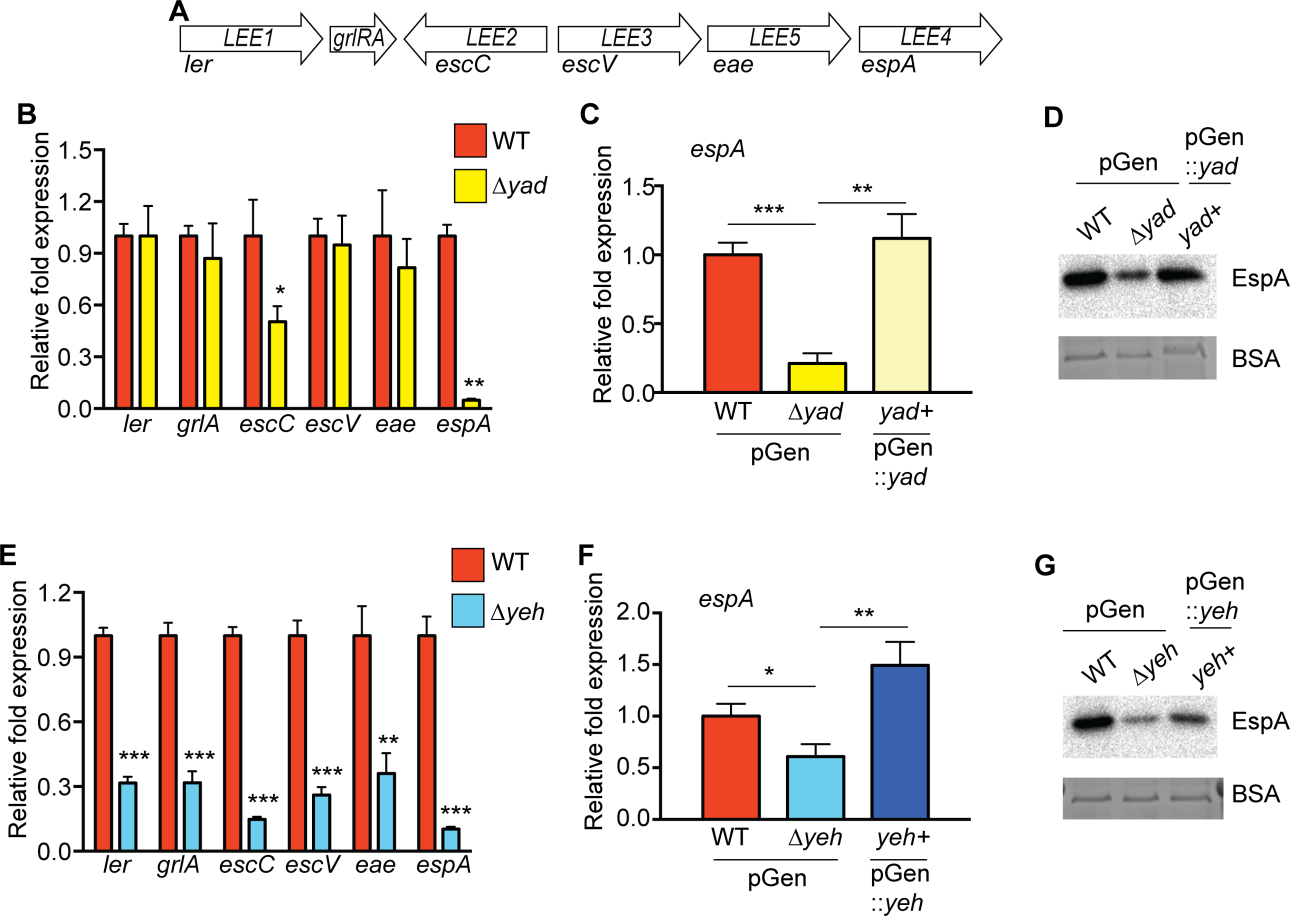
Yad and Yeh enhance LEE expression. (**A**) Schematic of the LEE. (**B**) RT-qPCR of LEE-encoded genes in WT and ∆*yad* strains. (**C**) RT-qPCR of *espA* in WT with empty vector, ∆*yad* with empty vector, *yad*+ (rescued with plasmid complementation of *yad*). (**D**) Representative Western blot of EspA in supernatants of WT with empty vector, ∆*yad* with empty vector, *yad*+ (rescued with plasmid complementation of *yad*). Bovine serum albumin (BSA) was added as a loading control. (**E**) RT-qPCR of LEE-encoded genes in WT and ∆*yeh* strains. (**F**) RT-qPCR of *espA* in WT with empty vector, ∆*yeh* with empty vector, *yeh*+ (rescued with plasmid complementation of *yeh*). (**G**) Representative Western blot of EspA in supernatants of WT with empty vector, ∆*yeh* with empty vector, *yeh*+ (rescued with plasmid complementation of *yeh*). BSA was added as a loading control. For panels **B**, **C**, **E**, and **F**, *n* = 3; error bars represent the geometric mean ± SD. *, *P* ≤ 0.05; **, *P* ≤ 0.005; ***, *P* ≤ 0.0005; ns, *P* > 0.05.

The LEE pathogenicity island contains 41 genes organized into five major operons (*LEE1, LEE2, LEE3, LEE5, LEE4*) that encode all of the T3SS structural components as well as effector proteins (12–14, 48–55) (Fig. 4A). The LEE also encodes regulatory proteins, including the transcriptional regulator Ler, as well as the regulators GrlA and GrlR that respectively promote or repress LEE expression (54, 56). Expression of the genes involved in T3S was among the most highly affected by the *yad* and *yeh* deletions (Fig. 3A and B). To examine Yad- and Yeh-dependent effects on LEE expression in more detail, we performed RT-qPCR and measured expression of one gene in each LEE operon. In the ∆*yad* strain, expression of *LEE2* and *LEE4* was decreased compared to WT (Fig. 4B). Furthermore, complementation of the ∆*yad* strain with a WT copy of the entire *yad* locus under control of the native promoter restored *espA*/EspA expression and secretion, respectively (Fig. 4C and D). In the ∆*yeh* strain, *ler* expression was significantly decreased compared to WT, and we measured a corresponding decrease in *LEE2-LEE4* (Fig. 4E). Plasmid complementation with the WT *yeh* locus (including the native promoter) rescued LEE expression (Fig. 4F and G).

**Fig 5:**
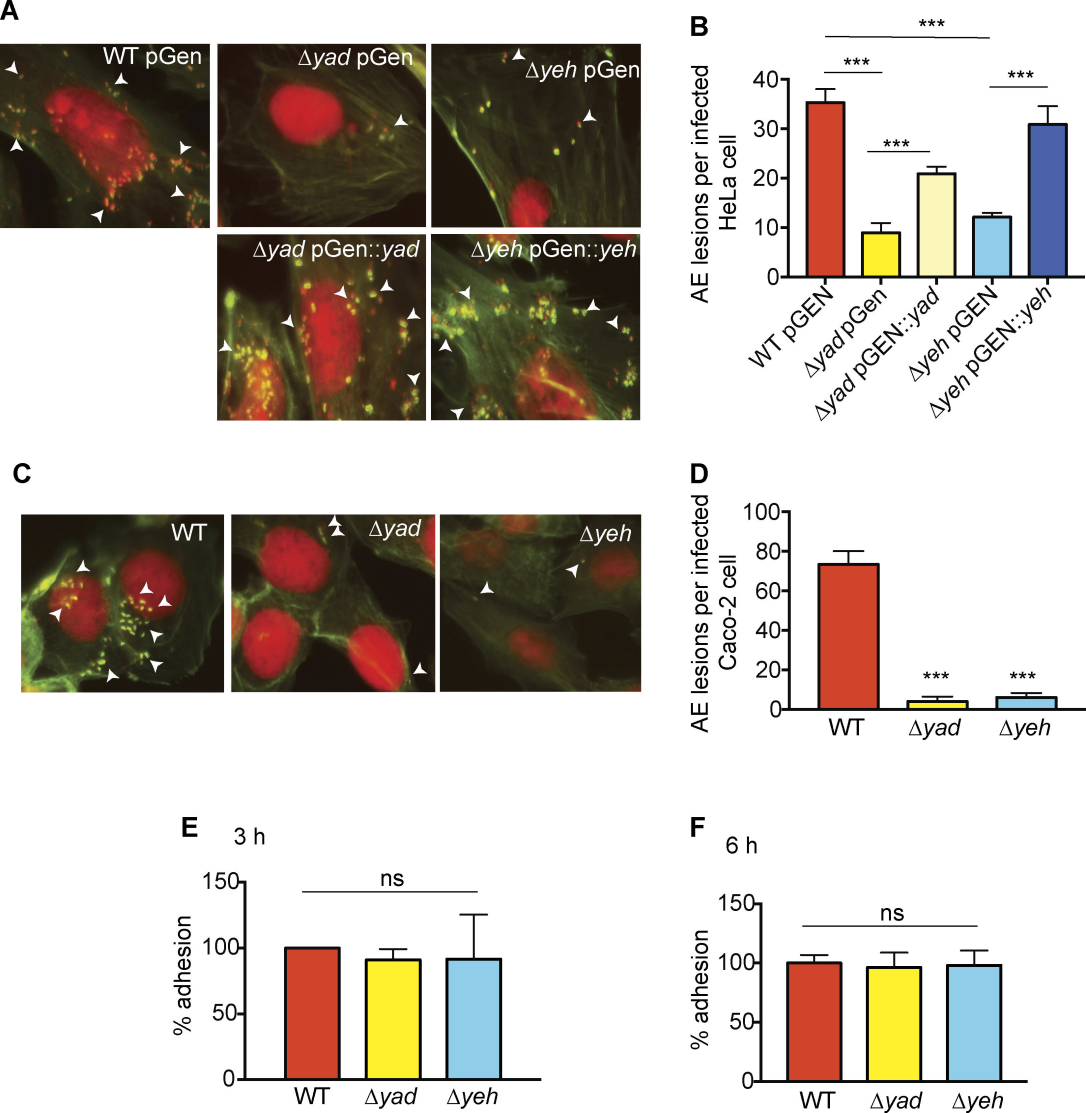
Yad and Yeh promote AE lesion formation. (**A**) Fluorescent actin staining (FAS) assay showing HeLa cells infected with WT EHEC, ∆*yad*, ∆*yeh*, or complemented strains as indicated. Epithelial cell nuclei and bacteria (red) and epithelial cell actin cytoskeleton (green) are shown. Arrows point to AE lesions that are visualized as punctate green structures associated with bacterial cells. (**B**) Quantification of panel **A**, *n* = 400 cells per strain. (**C**) FAS assay showing Caco-2 cells infected with WT EHEC, ∆*yad,* or ∆*yeh* strains as indicated. (**D**) Quantification of FAS assay. *n* = 400 cells per strain. (**E**) Percentages of adhering bacteria to HeLa cells at 3 h post infection. *n* = 9. (**F**) Percentages of adhering bacteria to HeLa cells at 6 h post infection. *n* = 9. Error bars represent the geometric mean ± SD. *, *P* ≤ 0.05; **, *P* ≤ 0.005; ***, *P* ≤ 0.0005; ns, *P* > 0.05.

LEE expression is required for AE lesion formation (57). Yad positively impacts expression of *LEE4*, which encodes the needle filament EspA and the translocon proteins EspBD. These secreted proteins mediate attachment to and pore formation in the host cell membrane and hence are required for effector delivery into host cells (58–61). Yeh enhances *ler* expression, which is the master regulator of the LEE (54, 62–64). Therefore, we reasoned that Yad and Yeh are important for AE lesion formation. To investigate this, we infected HeLa or Caco-2 cells with WT, the ∆*yad* strain, or the ∆*yeh* strain and then fixed and stained the cells to visualize AE lesions. In agreement with the gene expression studies, the ∆*yad* and ∆*yeh* strains formed significantly fewer AE lesions compared to WT on HeLa cells (Fig. 5A and B) and Caco-2 cells (Fig. 5C and D), and the defect in AE lesion formation could be partially or fully restored, respectively, in the *yad* or *yeh* complemented strains (Fig. 5A and B). EHEC colonization of the intestine is modeled to occur in distinct stages: transient, loose adherence mediated by fimbriae, followed by LEE-mediated intimate adherence (17, 65). Therefore, to better understand whether the defect in AE lesion formation in the ∆*yad* and ∆*yeh* strains was a result of a general decrease in bacterial attachment, we infected HeLa cells with WT EHEC, the ∆*yad* strain, or the ∆*yeh* strain and examined total adhering bacteria. All strains adhered at similar levels (Fig. 5E and F) strongly suggesting that Yad and Yeh promote AE lesion formation by influencing LEE expression. These data, together with data presented in Fig. 3 and 4, support an important and complex role for fimbriae in virulence, beyond adherence, as factors that direct gene expression.

### Yad and Yeh are required for maximal colonization of the mammalian GI tract

**Fig 6:**
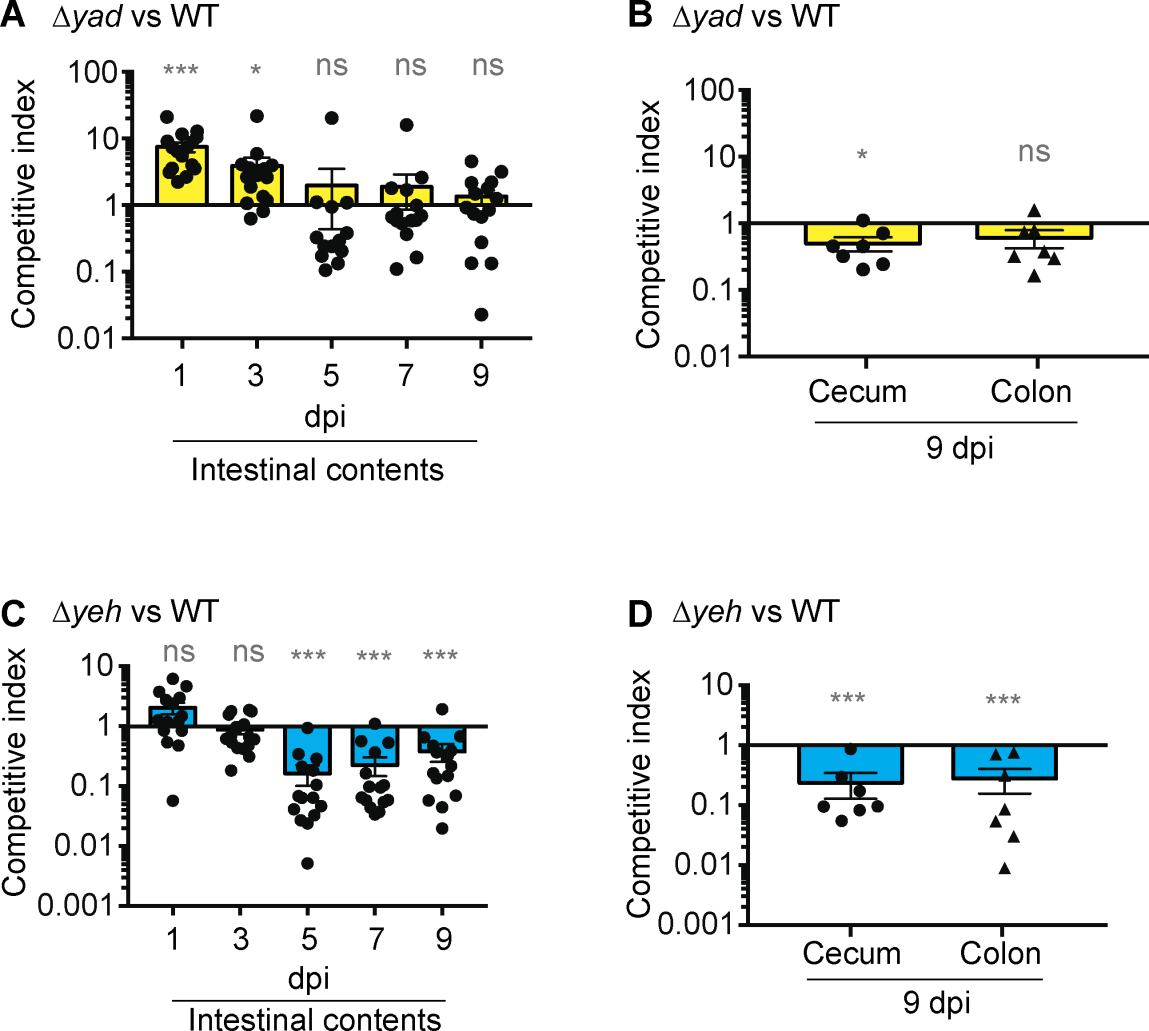
Yad and Yeh are required for robust colonization of the GI tract. (**A**) Competition assay between WT (∆*lacZ*) and Δ*yad* strains harvested from fecal samples at indicated time points. (**B**) Competition assay between WT (∆*lacZ*) and Δ*yad* strains harvested from the cecum or colon. (**C**) Competition assay between WT (∆*lacZ*) and Δ*yeh* strains harvested from fecal samples at indicated time points. (**D**) Competition assay between WT (∆*lacZ*) and Δ*yad* strains harvested from the cecum or colon. For these experiments, mice were orogastrically inoculated with 1:1 mixtures of indicated strains. Colony forming units (CFUs) were determined at indicated time points. Each point represents a competitive index. Horizontal lines represent the geometric mean value for each group (*n* = 7–16 animals). *, *P* ≤ 0.05; **, *P* ≤ 0.005; ***, *P* ≤ 0.0005; ns, *P* > 0.05, based on a Wilcoxon signed-rank test.

The important roles for Yad and Yeh in EHEC gene expression suggest these fimbriae may contribute to intestinal colonization. To test this idea, we performed competition experiments using streptomycin-treated mice, which is an established model to evaluate the relative colonization capacity of an *E. coli* strain (66, 67). For these assays, mice were orally infected with a 1:1 mixture of a ∆*lacZ* deletion strain, which served as the WT control (the *lacZ* deletion does not affect the ability of EHEC to colonize the GI tract [68]), and the ∆*yad* strain or the ∆*yeh* strain. At the start of infection (1 and 3 days post infection [dpi]), the ∆*yad* strain was recovered in higher numbers compared to WT. However, at 5–9 dpi, the ∆*yad* strain was recovered at lower levels from the intestinal contents compared to WT (Fig. 6A), and the differences in ratios of ∆*yad* to WT recovered at these time points were significantly decreased compared to 1 and 3 dpi (*P* ≤ 0.005). Moreover, the ∆*yad* strain was recovered at slightly lower levels than WT from the cecum and colon of infected mice (Fig. 6B). These findings suggest that Yad plays a role in EHEC GI colonization at later stages of infection.

Strikingly, the *yeh* deletion significantly impaired the ability of EHEC to colonize the GI tract. Beginning at 5 dpi, the ∆*yeh* strain was recovered at lower numbers from intestinal contents compared to WT, and this difference in fecal shedding continued through 9 dpi when the experiment was terminated (Fig. 6C). Furthermore, these data were consistent with numbers of WT and the ∆*yeh* strain recovered from the cecum and colon (Fig. 6D). These findings reveal that Yeh is required for robust EHEC intestinal colonization.

## DISCUSSION

Fimbriae often define the initial interactions between a bacterial pathogen and the host (19). In Gram-negative bacteria, five classes of adhesins have been identified, two of which are the type IV pili (T4P) and the CU fimbriae (69). The T4P span the inner and outer membranes of bacterial cells and expand and retract using energy from ATP hydrolysis upon surface interaction (biotic or abiotic). This dynamic property of T4P provides a mechanical stimulus that triggers multiple signaling cascades important for virulence (33–37, 70). In contrast, CU fimbriae are anchored only in the outer membrane, and although these fimbriae are able to extend to more than five times their original length (71, 72), retraction has not been documented. Moreover, the idea that CU fimbriae impact gene expression has mainly been examined in the context of controlling expression of other surface adhesins (39–45). For example, fimbrial expression in and of itself can affect expression of other surface-expressed proteins (39–43). For example, the *pap* fimbrial locus of uropathogenic *E. coli* encodes a transcriptional regulator PapB that controls expression of the *pap* locus (73). PapB also binds DNA within another fimbrial locus, *fim*, to inhibit phase-switching and thus prevent expression of the type 1 fimbriae (74). The *yad* and *yeh* loci do not encode predicted DNA-binding proteins (15, 16); however, an alternative mechanism of fimbrial cross-talk has been reported. In *Salmonella*, expression of the type 1 fimbriae (*fim*) results in decreased expression of a second fimbrial locus, termed *pef* (43). The underlying mechanism was shown to be binding of the 5′UTR of the *fim* mRNA to CsrA, thereby preventing CsrA from binding the *pef* mRNA and promoting Pef expression (43). In this example, the 5´UTR of the *fim* mRNA (in the absence of the *fim* coding sequence) was sufficient to regulate Pef expression.

Here, we demonstrated that two EHEC fimbriae, Yad and Yeh, impact expression of an extensive repertoire of genes, including known and putative adhesins, as well as T3SS and Shiga toxin production. Yad and Yeh are encoded by non-pathogenic and pathogenic *E. coli*. Previously, we reported that EA promotes expression of fimbriae in commensal *E. coli* (75). It is intriguing that Yad and Yeh may also influence commensal adaptation to the intestinal environment; however, this idea requires further investigation. Both Yad and Yeh promoted T3SS expression, whereas only Yad affected expression of Shiga toxin. Shiga toxin is encoded by a lysogenic phage and is released during bacterial cell lysis when EHEC encounters environmental stresses (46, 76). Therefore, Yad may limit Shiga toxin production under conditions in which LEE expression is favorable to enhance EHEC infection and replication within the colon. Regulation of EHEC virulence is complex and includes post-transcriptional regulation (46, 77–80), and the interplay of Yad and Yeh with other regulatory factors is not fully understood. We assessed gene expression in planktonically grown bacteria, suggesting that ability to influence gene expression is an intrinsic property of CU fimbriae. This idea is supported by previous work that showed that the long polar fimbriae also affects expression of approximately 120 genes in EHEC grown under similar conditions (81). Expression of CU fimbriae is often restricted *in vitro* (18, 19, 82). However, growth under *in vivo* conditions stimulates expression of a diverse repertoire of fimbriae (82), and our findings (Fig. 5) as well as reports by other groups (3, 17, 83–87) demonstrate that CU fimbriae play important roles in mammalian colonization. Thus, the precise expression of a fimbrial locus in response to host signals may provide an important cue for spatiotemporal control of traits important for colonization.

Because of the repertoire of diverse fimbriae within a bacterium, questions remain whether these fimbriae share redundant functions, whether they act synergistically to enhance infection, or whether these fimbriae play distinct roles (2). Our findings suggest that the answer is all of the above. For example, neither Yad nor Yeh was important for fimbrial-mediated adhesion to HeLa cells, which agrees with previous reports in which a fimbrial deletion did not impact adherence to epithelial cells and/or abiotic surfaces (17, 18, 88–90). Our findings also demonstrated that Yad positively impacted *yeh* expression, and Yeh positively impacted *yad* expression, indicating that positive cross-talk exists between the Yad and Yeh loci. Moreover, both fimbriae promoted LEE expression, suggesting a cooperative function for these fimbriae in enhancing EHEC intimate adherence to epithelial cells. Alternatively, it is possible that these fimbriae function in a feed-forward loop in which only one of these fimbriae primarily influences the observed changes in gene expression. Finally, transcriptomic analyses revealed differences in gene expression between the ∆*yad* and ∆*yeh* strains. Thus, a particular fimbria may influence how specific pathogenic steps are orchestrated and/or disease outcome.

CU fimbriae are common throughout the Proteobacteria (23). A paradigm in bacterial pathogenesis states that CU fimbriae function solely to attach bacteria to host tissue, and that this attachment enables subsequent pathogenic steps. In this model, after initiating attachment, fimbriae are passive in disease progression. Our findings indicate that the contributions of CU fimbriae to pathogenesis are more complex. Additional studies are required to fully understand the molecular mechanisms concerning how bacterial fimbriae influence gene expression. Nevertheless, these findings reveal an important role for fimbriae in bacterial physiology, as important determinants of gene expression, and provide fundamental insights into understanding interactions between bacteria and the host.

## MATERIALS AND METHODS

### Strains, plasmids, and general experimental details

Strains and plasmids used in this study are listed in Table S1. Oligonucleotide primers are listed in Table S2. For the RT-PCR experiments, strains were grown overnight in Luria-Bertani (LB) and then diluted 1:100 in M9 minimal and grown aerobically to an OD_600_ of 0.2. as previously described (22). For all other experiments, strains were grown overnight in LB and then diluted 1:100 in low-glucose Dulbecco’s modified Eagle medium (DMEM) (Invitrogen) and grown statically for 6 h under a 5% CO_2_ atmosphere at 37°C (21, 22). For all *in vitro* assays, the media was supplemented with 10 mM ethanolamine hydrochloride (EA; Sigma) and 150 nM vitamin B_12_ (cyanocobalamin; Sigma), which we previously reported promotes expression of EHEC fimbriae (21). Recombinant DNA and molecular biology techniques were performed using standard methods (91).

Gene deletions and mutations were generated using λ-red mutagenesis (92). To create the nonpolar deletions, the chloramphenicol or kanamycin cassettes were resolved using pCP20 (92). Final verifications were performed by Sanger sequencing.

The *yeh* and *yad* deletion strains were complemented with the entire *yeh* (*Z3276-Z3279*) or *yad* (*Z0146-Z0152*) fimbrial locus, including endogenous promoters. For this, strain LG01 was complemented with plasmid pLG01 to generate strain LG04, and strain LG02 was complemented with plasmid pLG02 to generate LG06. Plasmid pLG01 was generated with primer pairs, yeh_F_pGEN and yeh_R_pGEN, and pLG02 was generated with primer pairs, yad_F_pGEN and yad_R_pGEN using EHEC genomic DNA as a template. These products were digested with BamHI and EcorRI and inserted into pGEN-MCS (93) (Addgene MTA).

All experiments were repeated a minimum of two independent times with three biological replicates.

### Operon analysis by RT-PCR

RNA was extracted with the RiboPure Kit (Ambion) and was reverse transcribed and amplified by PCR (94).

### Microarray

Affymetrix 2.0 *E. coli* gene arrays were used to compare gene expression of strain 86-24 to that of ∆*yad* (LG02) *or* ∆*yeh* (LG01) as previously described (22). The RNA processing, labeling, hybridization, and slide-scanning procedures were performed as described in the Affymetrix Gene Expression technical manual. Data analyses from the array were performed as previously described (95). The Affymetrix GeneChip Command Console Software was used to obtain the output from scanning a single replicate of the Affymetrix GeneChip *E. coli* Genome 2.0 array for each of the biological conditions, following the manufacturer’s instructions. Data were normalized using robust multiarray analyses, and the resulting data were compared to determine genes whose expression was increased or decreased in response to the presence of *yad* or *yeh*.

### RT-qPCR

RNA from three biological replicates was extracted as described above. Primer amplification efficiency and template specificity of each of the primer pairs were validated, and reaction mixtures were prepared as described previously (22). RT-qPCR was performed in a one-step reaction using an ABI 7500FAST Sequence Detection System (Applied Biosystems). Data were collected using the ABI Sequence Detection 1.2 software (Applied Biosystems). Data were normalized to the levels of *rpoA* or 16S and analyzed using the comparative cycle threshold (*C_T_*) method (96). Unless indicated, the expression levels of target genes were compared using the relative quantification method (96). Real-time data are expressed as the changes in expression levels compared to the WT levels. For data shown in Fig. 2A, transcripts were measured using absolute quantification in which *C*_*T*_ values were evaluated against a standard curve generated using gDNA. Student’s *t*-test was performed to determine statistical significance.

### Adherence and fluorescent actin staining (FAS) assays

HeLa or Caco-2 cells were washed before infection and placed in low-glucose DMEM supplemented with EA and vitamin B_12_. The bacterial overnight (OVN) cultures were diluted to approximately 1.0 × 10^9^ colony forming units (CFU)/mL to infect the epithelial cells. Epithelial cells were incubated with the bacteria for 3 h or 6 h at 37°C in 5% CO_2_. In the 6 h assays, cells were washed and fresh medium was added after 3 h (97). To determine fimbrial-mediated adherence, after infection, epithelial cells were washed and lysed, and bacterial cells were plated to determine CFU/mL as previously described (12). The results of three independent experiments were averaged, and Student’s *t*-test was performed to determine statistical significance. To determine AE lesion formation, we performed FAS assays (97). Following infection, the coverslips were washed, permeabilized with 0.2% Triton X, and treated with fluorescein isothiocyanate-labeled phalloidin to visualize actin accumulation. Propidium iodide was added to stain the bacteria. Pedestal enumeration was performed for 400 infected cells per bacterial strain. A Student’s *t*-test was performed to determine statistical significance.

### Western blot analysis

For whole cell lysates, cells were collected by centrifugation, washed, and lysed under denaturing conditions. Samples were probed with rabbit polyclonal EspA antisera (Vanessa Sperandio) or DnaK (Thermo Fisher) or monoclonal antisera against Stx2a (Santa Cruz Biotechnology), RpoA (Santa Cruz Biotechnology), or FLAG (Sigma) and visualized with enhanced chemiluminescence (BioRad). Secreted proteins were collected as previously described (12). Secreted protein from culture supernatants was separated from bacterial cells using centrifugation and filtration. Samples were subjected to immunoblotting with antiserum to EspA and visualized as above. For the secreted protein analyses, 20 µg of bovine serum albumin was added to secreted protein samples as a loading control and visualized using Coomassie blue staining. For all Western blot experiments, a minimum of three independent experiments were performed.

### EMSAs

5′ biotinylated RNA probes were ordered from Integrated DNA Technologies (IDT). Samples were incubated for 30 min at 37°C in binding buffer. Following the addition of 5× RNA loading dye (50% glycerol, 0.1% bromophenol blue), samples were subjected to electrophoresis on 5% native Tris-borate-EDTA (TBE) gels using 1× TBE as running buffer. Electrophoresed samples were transferred by capillary action to Zeta-Probe membranes (BioRad) in 20× saline-sodium citrate (SSC). After UV crosslinking the membrane, the membranes were subjected to the Chemiluminescent Nucleic Acid Detection Module Kit (Thermo Scientific) according to the manufacturer’s protocol. EMSAs were visualized using a Gel Doc XR+ Gel Documentation System as previously described (79).

### Animal experiments

Mice (CD-1, 5 to 6 weeks old, male) were given streptomycin-treated water (5 g/L) 24 h prior to and throughout infection (98). Mice were inoculated with an equal number of 4 × 10^8^ CFU of the indicated strains (99). Fecal pellets were collected daily, weighed, and homogenized in phosphate-buffered saline (PBS). Bacterial numbers were enumerated by plating serial 10-fold dilutions of homogenates on MacConkey agar. For these assays, a ∆*lacZ* EHEC strain was used as WT. The *lacZ* deletion does not affect the ability of EHEC to colonize the GI tract (68, 100), but allows for differentiation between strains growing on MacConkey agar. The ratio of recovered WT to mutant bacteria was normalized by the ratio in the inoculum to determine the competitive index. Seven to 10 animals were used per group. A Wilcoxon signed-ranks test was used to determine statistical significance using 1.0 as a theoretical mean.

## Data Availability

The microarray data have been deposited in the National Center of Biotechnology Information Gene Expression Omnibus database (accession no. GSE100200).

## SUPPLEMENTAL MATERIAL

Table S1 msphere.00124-24-s0001.pdf Strains and plasmids used.

Table S2 msphere.00124-24-s0002.pdf Primers used.

ASM does not own the copyrights to Supplemental Material that may be linked to, or accessed through, an article. The authors have granted ASM a non-exclusive, world-wide license to publish the Supplemental Material files. Please contact the corresponding author directly for reuse.
