# Supplementary material for: The yad and yeh fimbrial loci influence gene expression and virulence in enterohemorrhagic Escherichia coli O157:H7
Source: mSphere. 2024 Jun 21;9(7):e00124-24. doi: 10.1128/msphere.00124-24 (PMC11287998; doi:10.1128/msphere.00124-24)
Supplement: Table S1 — Strains and plasmids used. [file msphere.00124-24-s0001.pdf]

Table S1. Strains and plasmids used in this study.

Bacterial strain	Description	Source
86-24	Wild-type EHEC strain (serotype O157:H7)	(1)
LG01	86-24 *yeh* mutant	This study
LG02	86-24 *yad* mutant	This study
BH21	86-24 *lacZ* mutant	(2)
LM01	86-24 with plasmid pGEN-MCS	This study
LM02	86-24 with plasmid pBAD24	This study
LG03	Δ*yeh* with plasmid pGEN-MCS	This study
LG04	Δ*yeh* with plasmid pLG01	This study
LG05	Δ*yad* with plasmid pGEN-MCS	This study
LG06	Δ*yad* with plasmid pLG02	This study
LG07	Δ*yeh* with plasmid pBAD24	This study
YehD-3xFLAG	86-24 with 3xFLAG inserted in frame with YehD	This study
csrA::*kan*	86-24 with *kan* insertion at codon 51 in *csrA*	This study
Plasmid name		
pKD3	pANTSλ derivative containing FRT-flanked chloramphenicol resistance	(3)
pKD46	λ red recombinase expression plasmid	(3)
pCP20	TS replication and thermal induction of FLP synthesis	(3)
pGEN-MCS	Cloning vector	(4)
pBAD24	Cloning vector	ATCC 87399
pLG01	*yeh* in pGEN-MCS	This study
pLG02	*yad* in pGEN-MCS	This study
pBAD/mychis A	Expression vector containing arabinose inducible promoter and Myc/His tag	Sigma
p*csrA*	pBAD/mychis A containing *csrA* inserted with	This study

KpnI and HindIII

REFERENCES

1. Griffin PM*, et al.* (1988) Illnesses associated with *Escherichia coli* O157:H7. *Ann. Intern. Med.* 109:705-712.
2. Pacheco AR*, et al.* (2012) Fucose sensing regulates bacterial intestinal colonization. *Nature* 492:113-117.
3. Datsenko KA & Wanner BL (2000) One-step inactivation of chromosomal genes in *Escherichia coli* K-12 using PCR products. *Proc. Natl. Acad. Sci.* 97(12):6640-6645.
4. Lane MC, Alteri CJ, Smith SN, & Mobley HLT (2007) Expression of flagella is coincident with uropathogenic *Escherichia coli* ascension to the upper urinary tract. *Proc. Natl. Acad. Sci.* 104:16669-16674.
