# Supplementary material for: The yad and yeh fimbrial loci influence gene expression and virulence in enterohemorrhagic Escherichia coli O157:H7
Source: mSphere. 2024 Jun 21;9(7):e00124-24. doi: 10.1128/msphere.00124-24 (PMC11287998; doi:10.1128/msphere.00124-24)
Supplement: Table S2 — Primers used. [file msphere.00124-24-s0002.pdf]

Table S2. Oligonucleotides used in this study.

yeh_LRF	ATCATGAAACATTCAATTATTGCTGTCGCTGTCTTATCTTCTGTATTTA TGTGTAGGCTGGAGCTGCTTC
yeh _LRR	TTCTTATTATTAGCCACTTGCTCATCTTGCTTGTTATTAATCGTATTTC ACATATGAATATCCTCCTTAG
yad_LRF	AGGATGCATGTAATGAAAAAAGCACTTCTCGCAGCCGCTCTGGTTAT GGCTTGTGTAGGCTGGAGCTGCTTC
yad _LRR	CGTTTTACTTATTCGTAGGTAAAGGAGAAGGTCGCGTTACCTGAAAAT GTTCCAACACCATATGAATATCCTCCTTAG
yeh_F_pGEN	CTAGGAATTCTTGCCAACACCGTTTTAAGCAT
yeh_R_pGEN	CTAGCCTAGGGATCAGCGACACCGACGGTA
yad_F_pGEN	CTAGGAATTCAGCACTTCATGCAAATAGATTAGGC
yad_R_pGEN	CTAGCCTAGGAGCAGAACTCTGGTGCGATG
yeh_pBAD_F	GGTACCGAGATACAGACTCTTAACAA
yeh_pBAD_R	AAGCTTTTATTATTAGCCACTTGCTC
ler_F	CGACCAGGTCTGCCC
ler_R	GCGCGGAACTCATC
grlA_F	CCGGTTGTTCCAGGACTTTC
grlA_R	TAAGCGCCTTGAGATTTTCATTT
escC_F	GCGTAAACTGGTCCGGTACGT
escC_R	TGCGGGTAGAGCTTTAAAGGCAAT
escV_F	TCGCCCCGTCCATTGA
escV_R	CGCTCCCGAGTGCAAAA
eae_F	GCTGGCCCTTGGTTTGATCA

eae_R	GCGGAGATGACTTCAGCACTT
espA_F	TCAGAATCGCAGCCTGAAAA
espA_R	CGAAGGATGAGGTGGTTAAGCT
stx2a_F	ACCCCACCGGGCAGTT
stx2a_R	GGTCAAAACGCGCCTGATA
rpoA_F	GCGCTCATCTTCTTCCGAAT
rpoA_R	CGCGGTCGTGGTTATGTG
16S_F	CAAGACCAAAGAGGGGGACC
16S_R	TTCCAGTGTGGCTGGTCATC
Z3278_F	CCACCGAATAGCCCAGAAGA
Z3278_R	TACCGGAGCAATACCAGCAG
Z3277_F	ATTGCTCGGCATTGAAGCAC
Z3277_R	CAATGTCGTACTGCCCAGGT
Z3276_F	GCGGGGGATACATTTACGCT
Z3276_R	TCCTTACTGTTCGCCGCAAT
Z0152_F	GGCGTGGTTACCCTGGATAC
Z0152_R	GGAGCACCTGGATCACACTC
Z0151_F	CTTTCCGCACACGCATCAAA
Z0151_R	GGCGTTGGGTTATTTGCCTG
Z0150_F	TCCGCCCGGTGAATTTGTTA
Z0150_R	AGAAGGGGTGCGTAAAGGTG
Z0149_F	ATCCGCGTTAATAGGCCTGG
Z0149_R	AGTTATCGTGCAGGTGGTGG
Z0148_F	AAACAGCGCCCAAATAGTGC
Z0148_R	AGTCCATACTTCCACAGCCG
Z0147_F	ATTGCCAGGTGCTACCGAATA
Z0147_R	GAAAAACGTCGGCATTGTGA
yeh/D_F	TGGCGGTGATTCAGTCAGTATT

yeh/D_R	ACCAACCGCGCCATCATA
yad_F	TCTGGGTGCTTGGTATGAAGTTAT
yad_R	TGTGGTGCCGCAGATGAA
ecp_F	CGTGGCTATCGAGGGTGACT
ecp_R	CTGGGTTAATGTGTTGGTGATAAGA
stc_F	CCAAGTATCGAAGTACTAGCATCAGAGT
stc_R	CCCTGGTCTCATTCTGGTCAA
csg_F	GCGTTGTCAATGGATTGCAA
csg_R	TCAGGTAACTGGCAAGCTTTTG
lpf1_F	TCTGTATTTGCTGCGGTTGGT
lpf1_R	GGTATCGCAATCTTCCAGTTTGA
stf _F	ACGGCCGAATCGTCAAAG
stf _R	CGTGACCCTCAAATGGAAATG
Sfm_F	TCTCAAACCTGATGGGAATAGCTT
Sfm_R	CGTGCAGAAAAATGAAGAACGT
ybg_F	CGCCCTGGCCATATCTCTAC
ybg_R	TTTAAAGTGGATTTCACCAGATCCT
ycb_F	GCAGATACCCGGTGGCATAT
ycb_R	TCCCTGGCCGCCTAAAC
sfa_F	ACGCCGTTTACGGTTTCTGT
sfa_R	GGTGGTGTTGATTGCCTGATC
fim (loc8) _F	TGCCAGACAATTTCCAACGA
im (loc8) _R	GTCCCTTTGCTGCATTCACTTA
lpf2_F	TGGTTTCGCCGCTAATGG
pf2_R	CACCGTCAGCGGACCAA
fim_F	TGCACAGTTTCCACCACCAA
fim_R	CCGGCAGACATCAGACTGAA
hcp_F	GCACTCACCGACATGCTACAA

hcp_R	CCAGCGCGCACAACTCT
nadR_F	TAGCTGATGCCAGCGGTATG
nadR_R	TCCGGTATGCAGTGGGTAGA
ecpR_F	ATTGCGGCCAGAAAGTCAGA
ecpR_R	GCGTATACCACGCCCCTAAT
chaB_F	TTCAACAGCGCATGGGATCA
chaB_R	CCACTTTATGCGCGGTTTCT
pyrI_F	TGCCCGAACAGCAACTGTAT
pyrI_R	GCGATATCATTGGCGCGTTT
fliA_F	GAACGCTATGACGCCCTACA
fliA_R	TCCAGTTGCCCTATTGCCTG
yra_F	GCGCTTATGCCTCCTCAGAA
yra_R	CTGGTTCACCGTAACATCATCAA
yedW_F	TTCACTCGCAATAACCGTTCTG
yedW_R	CGCGCAAGGAGTTTCAGTTAC
yehD_FLAG_fwd	TACAGTTAACTCTTCATTGGTTATGCGTGTTGCCCAGGATGACTACAA<br>AGACCATGACGG
yehD_FLAG_rev	AACTTACTGAAAAAACAAGATGATTATTTTAAATATTTAACATATGAAT<br>ATCCTCCTTAG
yeh_EMSA	CUUAUUAAGGAAUGCAUC
yeh_EMSA_mut	CUUAUUAACCAAUGCAUC
csrA_LR_fwd	GAAGTTTCTGTTCACCGTGAAGAGATCTACCAGCGTAGTGTAGGCTG<br>GAGCTGCTTC
csrA_LR_rev	GATTAGTAACTGGACTGCTGGGATTTTTCAGCCTGGATCATATGAATA<br>TCCTCCTTAG
CsrA_up	GGATACAGAGAGACCCGACT
CsrA_down	GTGCGTCTCACCGATAAAGA
CsrA_pBmh_fwd	CATGGTACCCTGATTCTGACTCGTCGAGTTG

CsrA_pBmh_rev	GTAAAGCTTGTAACTGGACTGCTGGGAT
